# Osteonecrosis of the jaw in a Crohn’s disease patient following a course of Bisphosphonate and Adalimumab therapy: a case report

**DOI:** 10.1186/1471-230X-14-6

**Published:** 2014-01-08

**Authors:** Raimund HM Preidl, Tobias Ebker, Martin Raithel, Falk Wehrhan, Friedrich W Neukam, Philipp Stockmann

**Affiliations:** 1 Department of Oral and Maxillofacial Surgery, University of Erlangen, Glückstraße 11, Erlangen 91054, Germany; 2 Department of Gastroenterology, Pneumology and Endocrinology, University of Erlangen, Erlangen, Germany

**Keywords:** Osteonecrosis of the jaw, Bisphosphonate, Adalimumab, Crohn’s disease

## Abstract

**Background:**

Bisphosphonates have a widespread indication for osteoporosis and are also applied in cancer patients with skeletal-related conditions. Bisphosphonate-associated osteonecrosis of the jaw (BRONJ) is a feared side effect which is hard to treat and often affects patient´s quality of life in an extensive manner. Adalimumab (Humira®), a fully human recombinant antibody specific for tumor necrosis factor- α, is approved for treatment in patients with Inflammatory Bowel Disease like ulcerative colitis or Crohn’s disease.

**Case presentation:**

In March 2013, a 36-year-old female presented with right-sided perimandibular swelling, recurrent facial pain and exposed necrotic bone after previous extraction of tooth 47. She had the medical history of Crohn’s disease for more than one decade with chronic active enterocolitis, fistula disease as well as previous oral manifestation and was currently treated with Adalimumab since September 2008. Due to steroid-induced osteoporosis, diagnosed in 2004, she received oral Bisphosphonates (Risedronate) from 2004 until 2007 followed by two infusions of Zoledronic acid in 2008 and 2009.

**Conclusion:**

This patient with a medical history of Crohn’s disease and gastrointestinal remission under Adalimumab therapy presented with osteonecrosis of the jaw after suspended oral and intravenous Bisphosphonate therapy implicating that the biologic therapy with an anti-TNF-α antibody might promote the manifestation of osteonecrosis and compromise oral healing capacity.

## Background

Bisphosphonates are primarily applied in patients with skeletal complications associated with osteoporosis as well as malignancy [1,2]. Bisphosphonate–associated osteonecrosis of the jaw (BRONJ), first described in 2003, poses a serious complication in patients currently or previously treated with Bisphosphonates and is associated with exposed bone in the maxillofacial region for at least 8 weeks without any radiotherapy of the jaw in the past [3,4]. The occurrence of BRONJ not only depends on the duration of the BP therapy but also varies between oral and intravenous application with far more cases reported after intravenous infusions with a cumulative incidence of 0,8%- 12% [5,6]. Although the pathomechanism is not yet completely understood, there are local risk factors like extraction of teeth, placement of dental implants, periapical surgery or dental abscesses going along with an increased incidence of osteonecrosis [7]. Beyond this, genetic and drug- related factors influence the appearance of BRONJ [8]. Clinically BRONJ presents as non-vital, exposed bone that might go along with inflammatory reactions due to secondary infection and therefore the gingival or mucosal tissue is usually sensitive to palpation. This process can aggravate to bone sequestration going along with acute osteomyelitis resulting in spreading and increased mobility of additional teeth [9]. Presumably, BRONJ is associated with infection and therefore immune-modulating drugs, as applied in patients with Crohn’s disease or rheumatoid arthritis, might be an important risk factor in the development of necrotic lesions in the jaw [10,11]. We already know that not only Bisphosphonates but also Denosumab or other biologicals are under suspicion to promote or even cause necrotic lesions in the jaw [12,13]. To our knowledge there is currently no published case of BRONJ in a patient with Crohn’s disease also affecting the oral cavity and treated with Adalimumab.

## Case presentation

**Figure 1:**
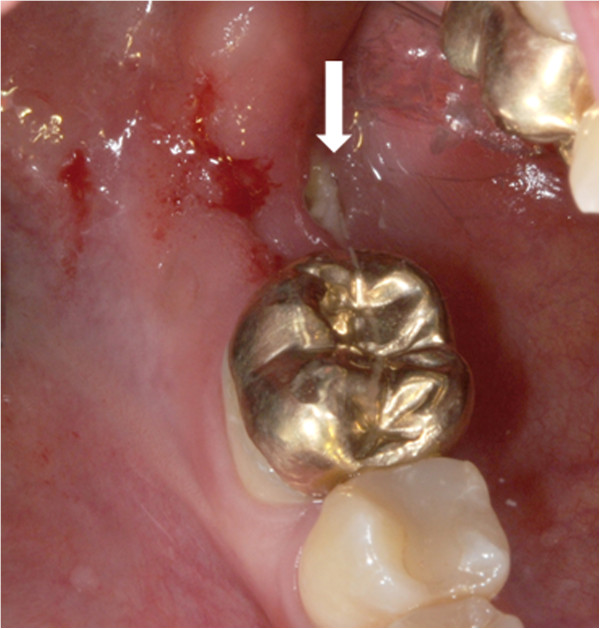
**Intraoperative situation with exposed necrotic bone lingual and crestal in the region of former tooth 47 (white arrow).** Inflammatory reaction within the necrotic region is also leading to local sugillations and bleedings as mucosal integrity seems to be disturbed. Crowned tooth 46 was removed within the operation.

**Figure 2:**
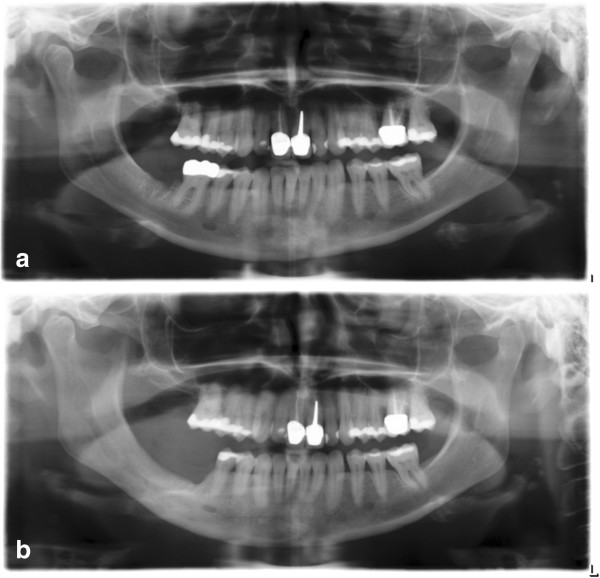
**Pre- and postoperative panoramic radiographs.** Preoperative radiograph **(a)** is showing a persistent extraction socket and hypersclerosis of the mandibular bone in the region 46/47 after extraction of 47 in January 2013. Postoperative panoramic radiograph **(b)** revealing a reduction of the mandibular bone height after repeated osteotomy and adequate ossification of the former extraction sockets.

A 36-year-old female presented in March 2013 with right-sided perimandibular swelling, cervical lymphadenopathy on the right side, dysphagia and pain on the lower face. In January 2013, tooth 47 was removed by the family dentist followed by episodes of recurrent pain during the following two months. On clinical investigation exposed bone surrounded by gingival inflammatory reaction was observed in the region of former tooth 47 (Figure 1). Panoramic radiograph revealed a persistent extraction socket of 47 (Figure 2a).

**Figure 3:**
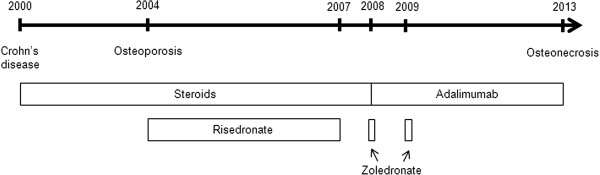
Drug regime since Crohn’s disease was diagnosed in 2000.

Her medical history revealed Crohn’s disease diagnosed in March 2000 affecting the colon, small intestine and stomach as well as aphthous oral lesions in the vestibulum in the years 2000 and 2001, fistula disease and extraintestinal manifestation with arthralgia. The patient was treated with varying doses of steroids together with 5-aminosalicylic (5-ASA) between 2000 and 2008 (Figure 3). In 2004 steroid-induced osteoporosis was diagnosed and associated with sintering fractures in the lower lumbar spine. Since then Calcium 500 mg and Cholecalciferol 1000 I.E. with concomitant oral Risedronate (Actonel®) 35 mg/week was applied until 2007 followed by two single 4 mg infusions of Zoledronic acid (Alcasta®) in 2008 and 2009. In September 2008, prednisolon and 5-ASA therapy was replaced by Adalimumab (Humira®) 40 mg/ 2 weeks for 3 years and later on monthly (cumulative dose approximately 3,6 g) leading to a total gastrointestinal remission regarding Crohn’s symptoms at the end of 2009 confirmed by endoscopy, histology and clinical disease activity in 2012. Between 2008 and 2013 recurrent events of psoriatic-like lesions, lupus-like erythrodermia and conjunctivitis were observed and treated with topically applied medication. She reported to be allergic to metals (gold, silver, nickel) and had no family history of Crohn’s disease.

**Figure 4:**
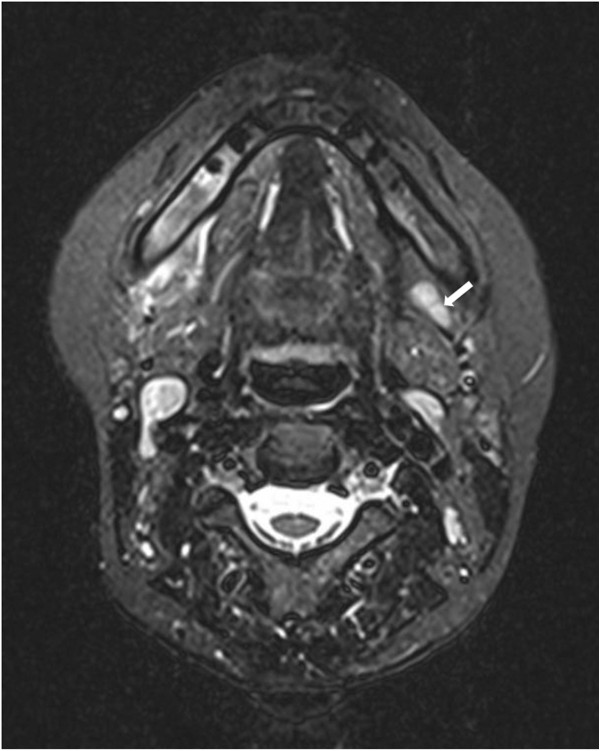
**Magnetic Resonance Imaging (MRI), t2 weighted, showing increased signalling in the jaw within the region of teeth 44 until 47 as well as in the ipsi- and contralateral lymph nodes (left side with white arrow).** Additionally, perimandibular soft tissue swelling is detectable on the affected side.

In March 2013, the patient was treated after previous MR-imaging revealed increased signalling in the jaw in the area of teeth 44 until 48 and additionally confirmed prominent lymph nodes on both sides of the submandibular region (Figure 4). Due to the fact that the mandibular bone was already exposed more than 8 weeks with no medical history of radiotherapy, BRONJ was diagnosed according to the AAOMS guidelines [6]. Surgical treatment consisted of osteotomy until healthy bone with bleeding from the surfaces was visible. Biopsies of bone were sent for pathological examination. Additionally, sharp edges of bone that can potentially traumatise the surrounding were removed. A mucoperiosteal flap was raised and primary wound closure was carried out without tension on the mucoperiosteal flap after incision of the periosteum with resorbable suture material (Vicryl 5–0, Ethicon, Norderstedt, Germany) [14]. Furthermore tooth 46 had to be removed because the necrotic bone affected the roots. Antibiotic therapy consisting of ampicillin/sulbactam (Ampicillin + Sulbactam-ratiopharm®, Ratiopharm, Ulm, Germany) 3 g/day and metronidazol (Metronidazol B. Braun®, B. Braun, Meisingen, Germany) 1,5 g/day and was administered intravenously for 8 days. Oral food intake was not allowed for 10 days and antiseptic mouth rinses (Hexetidin, 5-Amino-1,3-bis(2- ethylhexal) hexahydro-5-methylpyrimidin, Pfizer Pharma GmbH, Karlsruhe, Germany) was performed three times a day.

**Figure 5:**
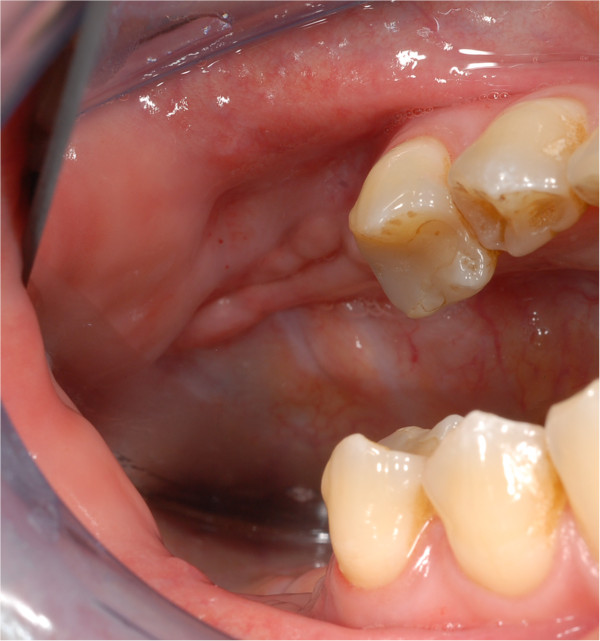
Postoperative situation in October 2013 showing recovery of the affected side after extraction of tooth 46 and repeated osteotomy.

Histological examination showed a granulomatous inflammatory reaction with necrotic, avital bone and actinomyces colonisation but no growth of mycobacteria or fungus and no multinucleated giant cells. Humira® therapy was stopped in March 2013 after consultation with the department of gastroenterology and due to the patient’s wish suffering from recurrent psoriatic-like lesions, lupus- like erythrodermia and conjunctivitis. 10 days after discharge the patient presented with adequate wound healing and mild clinical symptoms. During removal of the stitches bone got re-exposed again indicating a compromised mucosal healing. The patient was followed up weekly and received a second operative intervention at the beginning of April 2013 which improved the clinical situation (Figures 2b and 5).

## Discussion

Aminobisphosphonates are well established in the therapy of osteoporosis and metastatic cancer disease. Although BRONJ is a serious adverse event especially after intravenous administration, Aminobisphosphonates improve cancer control and increase long-term survival. This case report presents an extraordinary course of osteonecrosis of the jaw after mainly oral Bisphosphonate therapy and application of Adalimumab in a Crohn’s disease patient affecting almost the whole gastrointestinal tract. Bisphosphonates can cause gastrointestinal mucosal injuries and reduce bone remodelling by predominantly inhibiting osteoclasts via RANKL. Furthermore these drugs seem to have anti-angiogenic effects contributing to impaired wound-healing for example after tooth extraction [15,16].

Adalimumab, representing a fully humanised anti-TNF-α antibody, is an established biologic agent in the treatment of Crohn’s disease with proven clinical benefits [17]. Unfortunately there are already some reports about serious adverse effects like new-onset of multiple sclerosis, psoriasiform lesions, lupus like syndrome and infectious complications including sepsis in patients treated with the agent [18,19].

Cases of osteonecrosis in extracranial bones like the femur or the talus in patients with Inflammatory Bowel Disease and long lasting corticoid therapies have already been published [20,21]. Some even maintain that avascular osteonecrosis might be an extraintestinal manifestation of Inflammatory Bowel Disease although in some cases the interval between steroid treatment and avascular bone necrosis was quite extended [22,23]. However, in this report we present the first case of osteonecrosis in the jaw with the underlying history of Crohn’s disease being currently treated with Adalimumab (Humira®) which can be seen in a certain correlation to other cases demonstrating patients with osteonecrosis of the jaw and biological anti-TNF-α therapy with Infliximab [13].

The EXTEND trial revealed improved mucosal healing of gastrointestinal tissue under anti-TNF-α therapy [24], despite this it is still unclear whether anti-TNF-α treatment affects the oral mucosa and/or interferes with bone physiology, bone turnover, local immunity and wound repair on the long run. Pathomechanistically one could speculate at this stage that an inhibitory effect of Adalimumab on bone turnover might be mediated by a reduction of RANKL which could already be shown in patients with rheumatoid arthritis and anti-TNF-α therapy [25,26]. Interestingly osteonecrotic lesions in the jaw have also been reported within treatment regimes including Denosumab in cancer patients assuming that a blockade of receptor activator of nuclear factor-kappa-B (RANK) and receptor activator of nuclear factor-kappa-B- ligand (RANKL) interaction affects monocytic migration as well as osteoclast function comparable to Bisphosphonates [27]. We already know that Adalimumab induces apoptosis of activated human monocytes [28]. While apoptosis of disease-specific or reactive monocytes may be beneficial in idiopathic Inflammatory Bowel Disease, apoptosis of monocytes activated by infectious agents still present within the oral cavity may worsen wound healing, mucosal repair and bone repair of the jaw after necrosis. However, it still has to be investigated how bone metabolism and local infections surveillance in patients with Inflammatory Bowel Disease is influenced in the long run and how therapeutic strategies including biological substances lead to avascular bone necrosis in the jaw. Especially when considering already observed infectious complications due to immunosuppression associated with Adalimumab application it has to be investigated if osteonecrotic lesions might also occur because of spreading ongoing infections and/or have to be seen within the context of a certain innate immune dysfunction lacking defensin expression [29,30].

Regarding this case, on-going oral Crohn’s disease might be a confounder in terms of mucosal healing. Although the patient had no gastrointestinal symptoms including aphthous lesions in the oral cavity during Adalimumab- therapy, general oral mucosal healing capacities might be compromised.

## Conclusion

Patients with Inflammatory Bowel Disease and planned treatment with biological drugs like Adalimumab and a history of Bisphosphonate therapy should be also clinically investigated by a dentist before prescription of biologicals. Furthermore they should be monitored carefully with regard to osteonecrotic lesions of the jaw especially after dental procedures or within periodontal disease.

### Consent

Written informed consent was obtained from the patient for publication of this Case report and any accompanying images. A copy of the written consent is available for review by the Editor of this journal.

## Abbreviations

5-ASA: 5-aminosalicylic; BONJ: Bisphosphonate-associated osteonecrosis of the jaw; RANK: Receptor activator of nuclear factor-kappa-B; RANKL: Receptor activator of nuclear factor-kappa-B- ligand.

## Competing interests

The authors declare that they have no competing interests.

## Authors’ contributions

RP, TE, PS, FW examined and treated the patient and collected the data. RP, TE, MR, FWN discussed the case and data. RP, FW, MR, FWN and PS wrote the manuscript. All authors read and approved the final manuscript.

## Pre-publication history

The pre-publication history for this paper can be accessed here:

http://www.biomedcentral.com/1471-230X/14/6/prepub
